# Non-professional efferocytosis of *Salmonella*-infected intestinal epithelial cells in the neonatal host

**DOI:** 10.1084/jem.20231237

**Published:** 2024-02-02

**Authors:** Kaiyi Zhang, Urska Repnik, Nour Diab, Daniel Friske, Andreas Pütz, Alina Z. Bachmann, Narasimha Murthy Keshava Prasad Gubbi, Michael Hensel, Konrad U. Förstner, Alexander J. Westermann, Aline Dupont, Mathias W. Hornef

**Affiliations:** 1 Institute of Medical Microbiology, RWTH Aachen University Hospital, Aachen, Germany; 2 Department of Biology, Central Microscopy Unit, University of Kiel, Kiel, Germany; 3 Department of Biosciences, University of Oslo, Oslo, Norway; 4 Division of Microbiology, University of Osnabrück, Osnabrück, Germany; 5 Institute of Molecular Infection Biology, University of Würzburg, Würzburg, Germany; 6 Helmholtz Institute for RNA-based Infection Research, Helmholtz Centre for Infection Research, Würzburg, Germany; 7 Department of Microbiology, Biocentre, University of Würzburg, Würzburg, Germany

## Abstract

The intestinal epithelium is the first line of defense against enteric pathogens. Removal of infected cells by exfoliation prevents mucosal translocation and systemic infection in the adult host, but is less commonly observed in the neonatal intestine. Instead, here, we describe non-professional efferocytosis of *Salmonella*-infected enterocytes by neighboring epithelial cells in the neonatal intestine. Intestinal epithelial stem cell organoid cocultures of neonatal and adult cell monolayers with damaged enterocytes replicated this observation, confirmed the age-dependent ability of intestinal epithelial cells for efferocytosis, and identified the involvement of the “eat-me” signals and adaptors phosphatidylserine and C1q as well as the “eat-me” receptors integrin-αv (CD51) and CD36 in cellular uptake. Consistent with this, massive epithelial cell membrane protrusions and CD36 accumulation at the contact site with apoptotic cells were observed in the infected neonatal host in vivo. Efferocytosis of infected small intestinal enterocytes by neighboring epithelial cells may represent a previously unrecognized mechanism of neonatal antimicrobial host defense to maintain barrier integrity.

## Teaser

Shedding of infected enterocytes into the gut lumen is a hallmark of the adult mucosal antimicrobial response. In contrast, enterocyte shedding is rare in the neonatal intestine. Instead, we observe internalization and degradation of infected enterocytes by neighboring epithelial cells.

## Graphical Abstract

**Figure:**
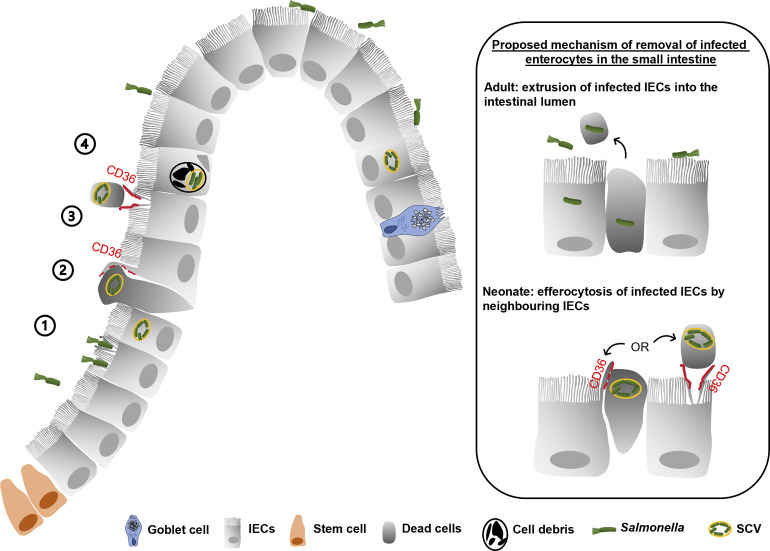

## Introduction

Professional efferocytosis is a fundamental biological process involved in tissue remodeling, homeostasis, and repair (Boada-Romero et al., 2020; Henson, 2017). Phagocytes engulf apoptotic cells to prevent inflammation by cellular components and to recycle metabolic substrates. In the adult intestine, macrophages or dendritic cells remove intestinal epithelial cells (IECs) by efferocytosis (Cummings et al., 2016). The functional relevance of this process is illustrated by the findings that gene polymorphisms associated with phagocyte efferocytosis are associated with chronic inflammatory bowel disease (Cummings et al., 2016) and that efferocytosis by dendritic cells is critical for controlling cytokine secretion and restoring homeostasis (Lee et al., 2017). Few reports have also linked efferocytosis to antimicrobial host defense (Capasso et al., 2016; Dallenga et al., 2017), although this aspect remains understudied in the intestine. Interestingly, in addition to phagocytes, stromal cells have also been described to be capable of efferocytosis then called non-professional efferocytosis. Efferocytosis by epithelial cells has been identified in the lung, mammary gland, liver, kidney, and retinal pigment epithelium (Davies et al., 2018; Juncadella et al., 2013; Monks et al., 2005; Ismail et al., 2018; Yu et al., 2019).

Here, we observed non-professional efferocytosis of *S.* Typhimurium-infected small intestinal enterocytes by neighboring epithelial cells in the neonatal host in vivo and characterized this process by immunofluorescence imaging and electron microscopy. Using transcriptomic profiling of isolated primary IECs and coculture of neonatal and adult small intestinal epithelial stem cell organoids with injured enterocytes, we confirmed the age-dependent propensity for non-professional efferocytosis and identified the expression and contribution of specific “eat-me” signals and receptors. Our results demonstrate non-professional efferocytosis in the context of enteric infection and suggest that efferocytosis at the intestinal epithelium contributes to the antimicrobial host defense in the neonatal gut.

## Results and discussion

**Figure 1.**
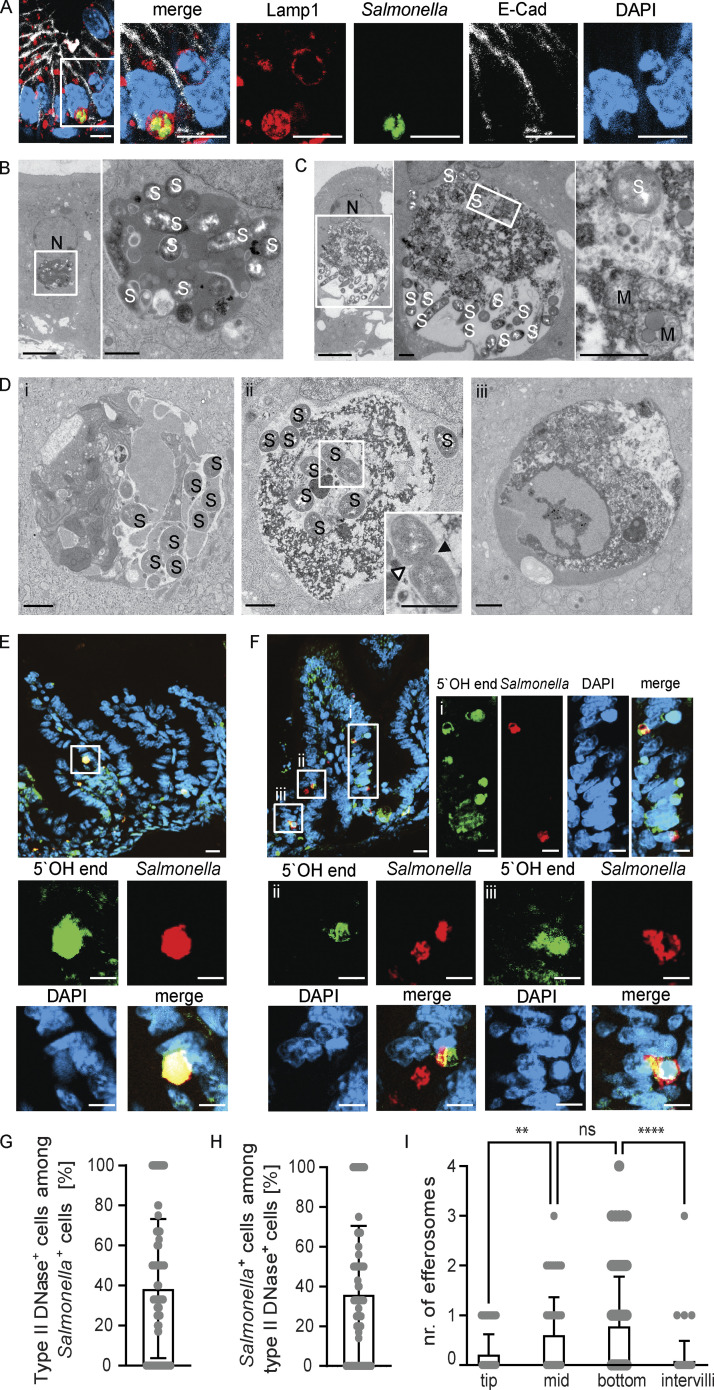
**Non-professional efferocytosis in the intestine of infected neonatal mice. (A)** Immunostaining for Lamp1 (red) and *S.* Typhimurium (GFP, green) in *S.* Typhimurium–infected neonatal small intestinal tissue sections at day 4 p.i. Counterstaining with E-cadherin (white) and DAPI (blue). Bar, 10 μm. **(B–D)** Transmission electron microscopy (TEM) of the small intestinal epithelium at day 4 p.i. **(B)** Intraepithelial SCV-like endosome with *S.* Typhimurium but without cell debris. The white square in the left panel indicates the area that is magnified in the right panel. N, nucleus; S, *S.* Typhimurium. Bar, 5 μm (left) and 1 μm (right panel). **(C)** Intraepithelial *Salmonella* containing endosome with cell debris likely derived from efferocytosis. The white square indicates the area that is magnified in the panel on the right. N, nucleus; M, mitochondria; S, *S.* Typhimurium. Bar, 5 μm (left) and 1 μm (middle and right panel). **(D)** Small intestinal epithelium illustrating intraepithelial endosomes containing *S.* Typhimurium and cell debris (i and ii) or only cell debris (iii). Arrowheads in the inset in panel ii indicate the growing septum of a dividing *S.* Typhimurium. S, *S.* Typhimurium. Bar, 1 μm. **(E and F)** Staining for 5′OH DNA strand ends as a sign of DNase type II activity (green) and *S.* Typhimurium (GFP, red) in small intestinal tissue sections at day 4 p.i. Counterstaining with DAPI (blue). Bar, 20 μm (overview image) and 10 μm (single-color channels). **(G and H)** Percentage of (G) type II DNAse (efferosome)-positive cells among all *S.* Typhimurium–positive IECs and (H) *S.* Typhimurium–positive cells among all type II DNAse (efferosome)-positive IECs. 40 image fields with the size of 312.35 × 250.61 µm of small intestinal tissue sections obtained at day 4 p.i. from three individual *S.* Typhimurium–infected neonatal animals were analyzed. **(I)** Quantitative evaluation of the number of type II DNAse (efferosome)-positive IECs at different regions of the epithelium along the crypt–villus axis (tip, middle, bottom, intervillus region). 68 villi from three individual *S.* Typhimurium–infected neonatal animals at day 4 p.i. were analyzed. One-way ANOVA Kruskal–Wallis test with Dunn’s post-test. **, P < 0.01; ****, P < 0.0001; ns, non-significant.

**Figure S1.**
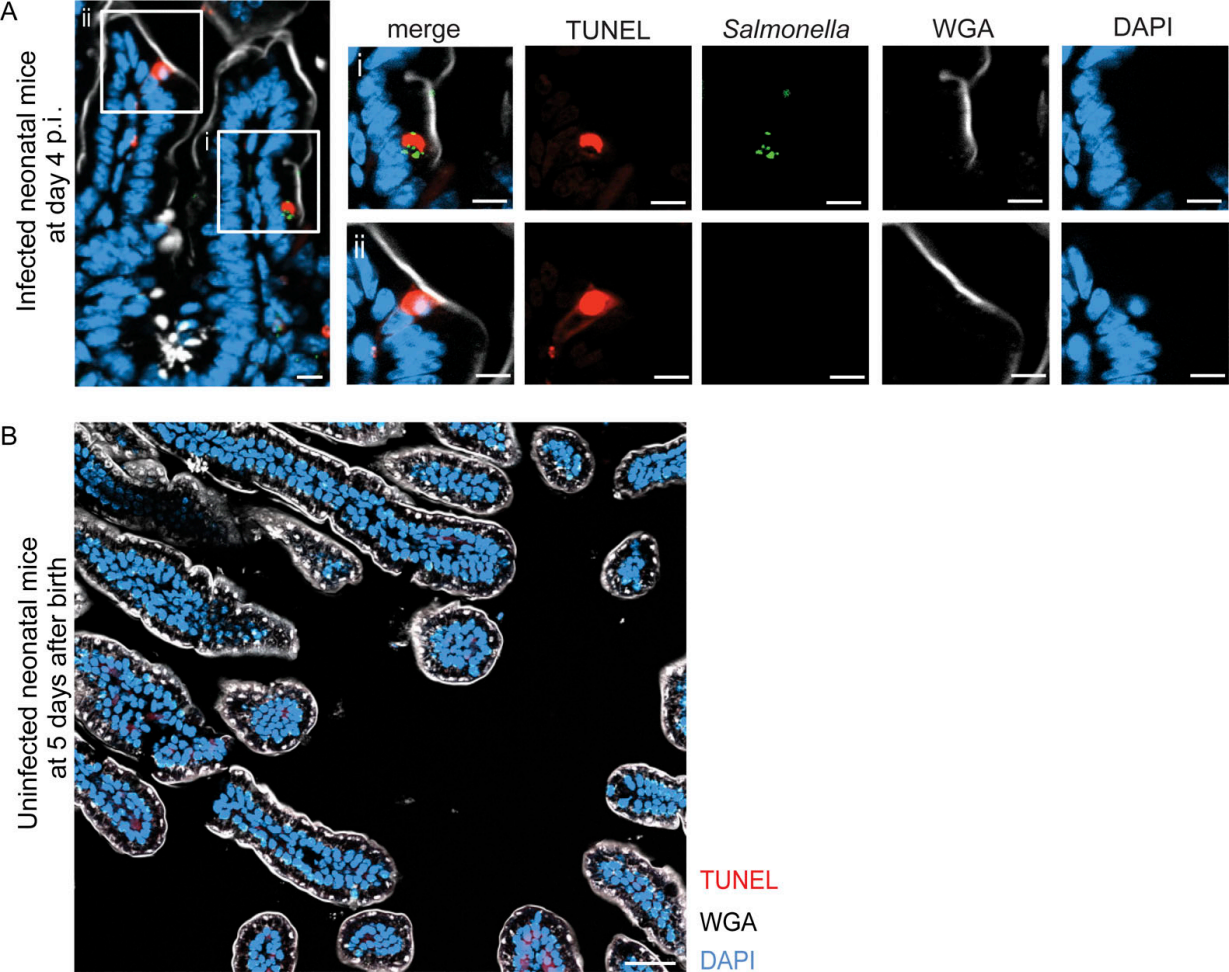
**TUNEL-positive epithelial cells in the intestine of healthy and *S.* Typhimurium–infected neonatal mice. (A)** Immunostaining for TUNEL (red) and *S.* Typhimurium (GFP, green) in small intestinal tissue sections at day 4 p.i. Counterstaining with WGA (white) and DAPI (blue). Bar, 10 μm. **(B)** Immunostaining for TUNEL (red) in a small intestinal tissue section of a 5-day-old healthy C57BL/6 mouse. Counterstaining with WGA (white) and DAPI (blue). Bar, 50 μm.

We have previously reported that enterocyte invasion and intraepithelial proliferation were readily observed at day 4 post infection (p.i.) in mice infected as 1-day-old newborns with 10^2^ CFU *Salmonella enterica* subsp. *enterica* sv. Typhimurium (*S.* Typhimurium; Zhang et al., 2014). Consistent with our previous report, intraepithelial *S.* Typhimurium–positive endosomes stained positive for the *Salmonella*-containing vacuole (SCV) marker lysosomal-associated membrane protein (LAMP)1 (Zhang et al., 2018; Fig. 1 A). On closer analysis of the small intestinal epithelium of *S.* Typhimurium–infected neonatal mice, we detected additional intraepithelial LAMP1-positive endosomes containing large amounts of DAPI-positive chromatin. GFP-positive *S.* Typhimurium was detected in some, but not all, of these chromatin-containing endosomes. TUNEL staining as a late cell death marker of the same tissue sections revealed TUNEL-positive material in IEC compartments adjacent to the nucleus, which may or may not contain detectable *S.* Typhimurium (Fig. S1 A). These TUNEL-positive cell compartments were detected in the intestinal epithelium of infected newborn mice, but not in age-matched healthy controls (Fig. S1 B). The two types of *Salmonella*-containing compartments were confirmed by ultrastructural analysis. Previously described SCV-like, membrane-enclosed *Salmonella*-containing compartments were characterized by amorphous, electron-dense luminal contents (Fig. 1 B; Zhang et al., 2014). In addition, endosomes were observed with cargo indicative of cellular components such as chromatin or mitochondria (Fig. 1, C and D). The cell debris cargo showed varying degrees of degradation, suggesting that it was derived by efferocytosis. Again, *S.* Typhimurium was observed in some, but not all, endosomal structures containing cell debris (Fig. 1 D). These results suggest that neonatal epithelial cells act as non-professional phagocytic cells and internalize cell debris under conditions of infection.

**Figure S2.**
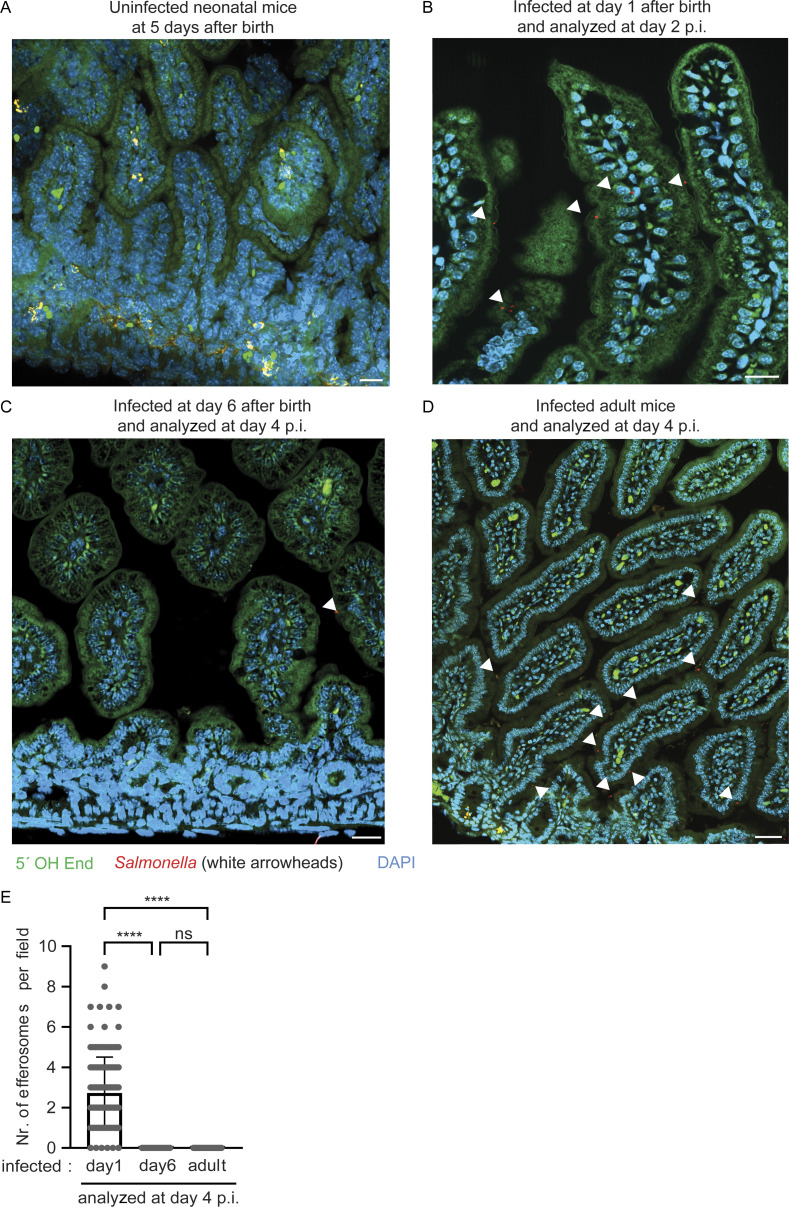
**DNAse type II activity positive epithelial cells in the intestine of infected mice of various age. (A–D)** Staining for 5′OH DNA strand ends as a sign of DNase type II activity (green) and immunostaining for *S.* Typhimurium (red, highlighted by white arrowheads) in small intestinal tissue sections of (A) 5-day-old healthy C57BL/6 mice, (B) 1-day-old mice orally infected with *S.* Typhimurium at day 2 p.i., as well as (C) 6-day-old mice and (D) adult mice infected with *S.* Typhimurium at day 4 p.i. Representative images are shown. Counterstaining with DAPI (blue). Bar, 20 μm (A and C) and 50 µm (B). **(E)** Total number of efferosomes was quantified in at least 16 image fields with the size of 312.35 × 250.61 µm (for neonates) or the size of 624.70 × 501.22 µm (for adults) of small intestinal tissue sections from three or four individual *S.* Typhimurium–infected neonatal and adult animals at day 4 p.i. One-way ANOVA Kruskal–Wallis test with Dunn’s post-test. ****, P < 0.0001; ns, non-significant.

Staining for 5′-hydroxyl (5′OH) DNA double-strand ends confirmed the presence of lysosome-derived DNAse II activity, characteristic of efferosomes, within the detected endosomal structures in the newborn mice at day 4 p.i. (Weir, 1993; Minchew and Didenko, 2011; Fig. 1, E and F). Collectively, ∼40% of *S.* Typhimurium–positive enterocytes also stained positive for DNAse II activity (Fig. 1 G). Conversely, ∼35% of DNAse II activity–positive enterocytes contained detectable intraepithelial *S.* Typhimurium (Fig. 1 H). Most DNAse II activity–positive efferosomes were observed at the mid and lower regions of the villus (Fig. 1 I), where it was previously reported that most *S.* Typhimurium–infected enterocytes reside (Zhang et al., 2014). Notably, no DNAse II activity–positive enterocytes were detected in healthy age-matched (5-day-old) control animals (Fig. S2 A) or very early (day 2) after infection of 1-day-old neonatal mice (Fig. S2 B). Also, enterocyte efferosomes were not detected in 10-day-old or adult *S*. Typhimurium–infected mice (Fig. S2, C–E). Thus, non-professional efferocytosis was only detected during the postnatal period in *S.* Typhimurium–infected animals, increased during the course of the infection, and included internalization of both *S.* Typhimurium–positive and –negative epithelial cells by neighboring enterocytes.

**Figure 2.**
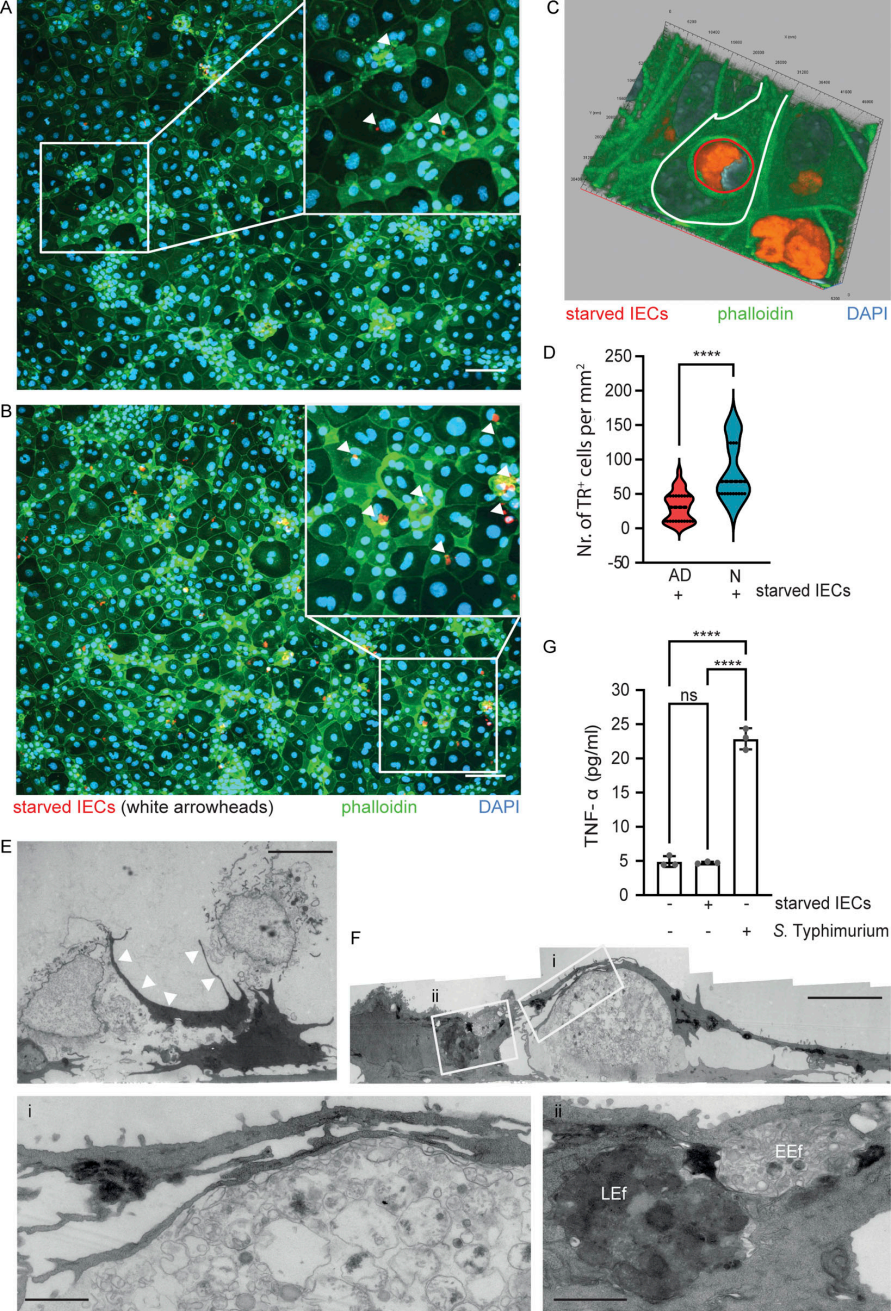
**Age-dependent propensity of IECs for efferocytosis. (A and B)** Immunostaining of intestinal epithelial stem cell organoid–derived cell monolayers generated from adult (A) or neonatal (B) small intestinal tissue 2 h after coculture with growth factor–starved tdTomato expressing IECs (starved IECs, red) derived from adult stem cell organoids. White squares indicate the area that is magnified; white arrowheads in the magnified image highlight intracellular tdTomato (red)-positive cell material. Counterstaining with phalloidin (green) and DAPI (blue). Bar, 100 μm. **(C)** Three-dimensional image reconstruction to illustrate the dtTomato-positive starved IEC (red, highlighted with a red line) engulfed by a neonatal stem cell organoid–derived IEC (highlighted with a white line). Counterstaining with phalloidin (green) and DAPI (blue). **(D)** Quantitative analysis of the number of internalization events of tdTomato-positive growth factor–starved cells/cell material (TR^+^ cells) by intestinal epithelial stem cell organoid–derived cell monolayers generated from neonatal (N) or adult (AD) small intestines per mm^2^. 30–49 image fields with the size of 1.25 × 1.00 mm obtained from two to three independent experiments were analyzed. Mann–Whitney U test. ****, P < 0.0001. **(E and F)** TEM images of a neonatal intestinal epithelial stem cell organoid–derived cell monolayer co-cultured with growth factor–starved intestinal epithelial stem cell organoid cells (starved IECs) for 2 h. **(E)** White arrowheads illustrate the membrane extensions of monolayer cells directed toward the starved IECs. Bar, 5 µm. **(F)** The white squares indicate the areas that are magnified in panels i and ii. Three stages of efferocytosis: uptake of cell debris (magnified in i), endosomal compartments with loosely packed cargo suggestive of an EEf, and with electron-dense cargo suggestive of an LEf (magnified in ii). Bar, 5 µm (panels i and ii, 1 µm). **(G)** TNF-α (pg/ml) in the cell culture supernatant of neonatal intestinal epithelial stem cell organoid–derived cell monolayers left untreated, stimulated with *S.* Typhimurium (MOI 10:1) or exposed to growth factor–starved intestinal epithelial stem cell organoid cells (starved IECs) for 2 h. Two independent experiments with three replicates were analyzed, the graph shows one representative experiment. One-way ANOVA Kruskal–Wallis test with Dunn’s post-test. ****, P < 0.0001; ns, non-significant.

**Figure S3.**
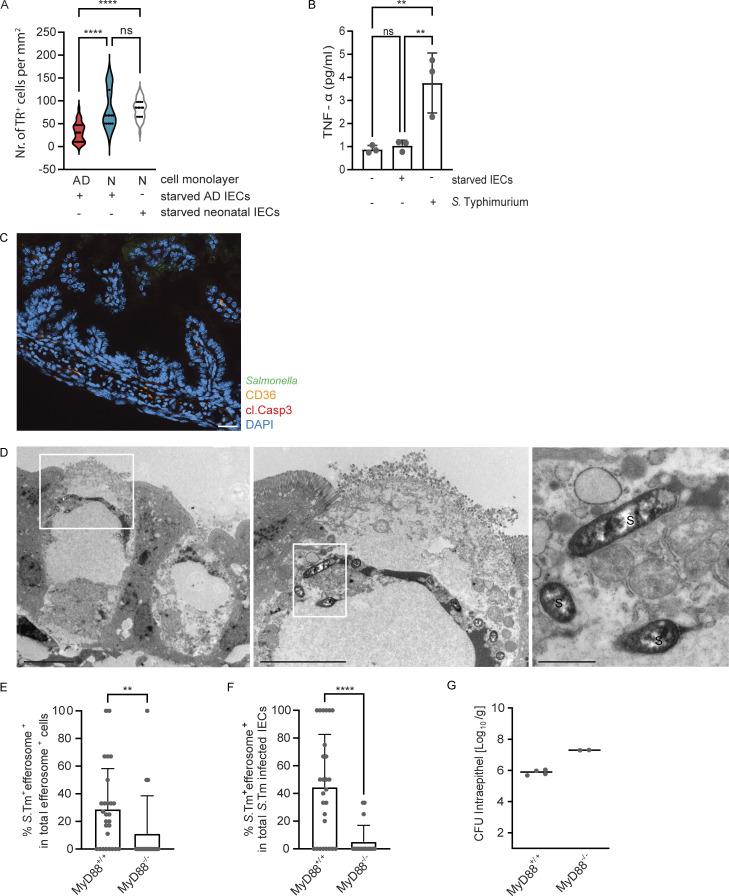
**The influence of age and MyD88 signaling for enterocyte efferocytosis in the stem cell organoid co-culture model. (A)** Comparative quantitative analysis of the number of internalization events of tdTomato-positive growth factor–starved cells/cell material (starved IECs) derived from adult stem cell organoids (starved adult IECs) or neonatal stem cell organoids (starved neonatal IECs) by intestinal epithelial stem cell organoid–derived cell monolayers generated from neonatal (N) or adult (AD) small intestines per mm^2^. 30–49 images (1.25 × 1.00 mm) obtained from two to three independent experiments were analyzed. The data for cell monolayers exposed to starved adult IECs are identical to Fig. 2 D. One-way ANOVA Kruskal–Wallis test with Dunn’s post-test. ****, P < 0.0001; ns, non-significant. **(B)** TNF-α (pg/ml) in the cell culture supernatant of adult intestinal epithelial stem cell organoid–derived cell monolayers left untreated, stimulated with *S.* Typhimurium (MOI 10:1), or exposed to growth factor–starved intestinal epithelial stem cell organoid cells (starved IECs) for 2 h. Two independent experiments with three replicates were analyzed; the graph shows one representative experiment. One-way ANOVA Kruskal–Wallis test with Dunn’s post-test. **, P < 0.01; ns, non-significant. **(C)** Immunostaining for CD36 (orange), cleaved caspase 3 (cl. Casp3, red) and *S.* Typhimurium (green) in small intestinal tissue sections of healthy 5-day-old mice. Counterstaining with DAPI (blue). Bar, 20 μm. A representative image is shown. **(D)** TEM images of an *S.* Typhimurium–infected IEC with signs of reduced viability. The white squares indicate areas that are displayed at higher resolution in adjacent panels on the right. Microvilli at the cell surface are vesiculated, and the cell cytoplasm appears vacuolized and extracted. The electron-dense, amorphous material represents the luminal contents of the SCV-like endosome. Bacteria are visible inside the SCV in the left cell. S, *S.* Typhimurium. Bar, 5 µm (left and middle panel), 1 µm (right panel). **(E–G)** 1-day-old MyD88^+/+^ and MyD88^−/−^ mice were orally infected with 10^2^ CFU *S.* Typhimurium (*S.* Tm). Small intestinal tissue sections at day 4 p.i. were used to quantify the percentage of (E) *S.* Typhimurium–positive cells among all efferosome-positive IECs and (F) *S.* Typhimurium–positive efferosome positive cells among all *S.* Typhimurium–positive IECs. 18–27 image fields with the size of 312.35 × 250.38 µm of small intestinal tissue sections obtained at day 4 p.i. from three to five individual *S.* Typhimurium–infected neonatal animals were analyzed. Mann–Whitney U test. **, P < 0.01; ****, P < 0.0001. **(G)** *Salmonella* organ load in isolated gentamicin-treated enterocytes from MyD88^+/+^ and MyD88^−/−^ mice at day 4 p.i. (*n* = 2–4).

Next, we established an intestinal epithelial stem cell organoid co-culture model to investigate the age-dependent phenotype and to analyze the mechanism of neonatal enterocyte efferocytosis. Spherical intestinal epithelial stem cell organoids were generated from primary intestinal crypt cells isolated from 1-day-old neonatal or 6-wk-old adult mice, trypsinized, and grown as cell monolayers to compare their propensity for efferocytosis (Sato et al., 2009; Sanman et al., 2020). In addition, spheroid stem cell organoids were generated from adult mice carrying the Rosa^mT/mG^ locus and ubiquitously expressing a membrane-bound form of the red fluorophore tdTomato. After growth, tdTomato-positive intestinal epithelial stem cell organoids were deprived of the essential growth factors R-spondin and noggin, trypsinized, and the starved cells were added to the neonatal or adult stem cell organoid–derived cell monolayers. After 2 h of co-cultivation, cell monolayers were washed and stained with phalloidin and DAPI. Fluorescence images were generated and analyzed to quantify the number of internalization events of tdTomato-positive cell material. tdTomato-positive material was detected in both adult and neonatal intestinal epithelial stem cell organoid–derived cells (Fig. 2, A and B) and was often associated with DAPI-positive chromatin material, consistent with our observation in vivo (Fig. 2 C). Importantly, neonatal stem cell organoid–derived cell monolayers harbored a significantly higher number of tdTomato-positive endosomes compared with adult stem cell organoid–derived cell monolayers (Fig. 2 D). In contrast, the developmental age (neonatal versus adult) of the growth factor–deprived starved tdTomato-positive IECs had no significant effect on the efferocytosis efficacy of neonatal stem cell organoid–derived cell monolayers (Fig. S3 A). Ultrastructural analysis of neonatal stem cell organoid co-cultures confirmed these findings. Neonatal stem cell organoid–derived enterocytes formed extensive pseudopodia to internalize cellular debris (Fig. 2 E). Furthermore, different stages of efferosome maturation were observed in stem cell organoid–derived enterocytes with different degrees of condensation of the cell debris cargo (early [EEf] and late [LEf] efferosomes) in analogy to phagosome maturation (Fig. 2 F; Vieira et al., 2002). Finally, exposure to live *S.* Typhimurium, but not starved enterocytes, induced a significant stimulation and release of TNF by neonatal and adult stem cell organoid–derived cell monolayers (Fig. 2 G and Fig. S3 B). These results illustrate the propensity of neonatal enterocytes for non-professional immunosilent efferocytosis.

**Figure 3.**
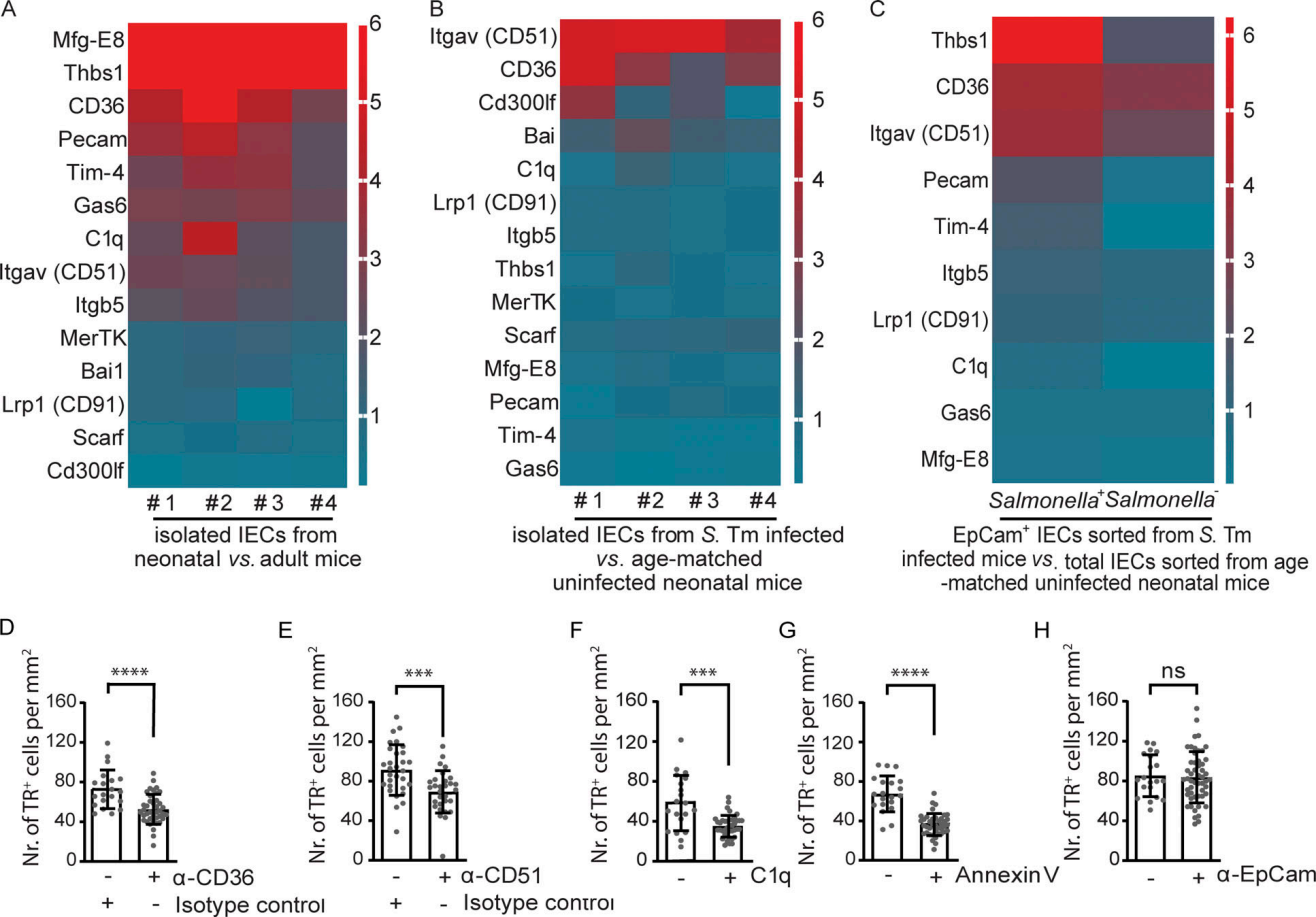
**Eat-me signals and receptors involved in infection-induced non-professional efferocytosis. (A and B)** Heat map of the fold increased expression of selected eat-me receptor, mediator, and signal genes in total isolated IECs from healthy neonatal (day 3 after birth) versus healthy adult (day 21 after birth) mice (A) and neonatal 1-day-old mice infected with 10^2^ CFU *S.* Typhimurium (*S.* Tm) at day 4 p.i. versus healthy age-matched uninfected neonatal mice (B). *n* = 4. **(C)** Heat map of the fold increased expression of selected eat-me receptor, mediator, and signal genes in flow cytometrically sorted *Salmonella*-positive (Salmonella^+^) and *Salmonella*-negative (Salmonella^−^) EpCam^+^ IECs obtained from infected neonatal mice versus total isolated IECs obtained from age-matched uninfected mice. 1-day-old mice were infected with 10^2^ CFU constitutively mCherry expressing *S.* Typhimurium. Total small IECs were isolated at day 4 p.i. and sorted by flow cytometry to obtain EpCam^+^ CD45^−^ *Salmonella*-positive (mCherry^+^) and EpCam^+^ CD45^−^ *Salmonella*-negative (mCherry^−^) cells. Total RNA of the described cell populations was obtained from a total of 49 neonatal animals from 7 litters. RNA samples from the same population were pooled into one sample for analysis, *n* = 1. **(D–H)** Identification of eat-me receptors, mediators, and signals involved in non-professional efferocytosis. Neonatal stem cell organoid cell monolayers were preincubated with an anti-CD36 antibody or an isotype control antibody (D), an anti-integrin-αv (CD51) antibody or an isotype control antibody (E), or an anti-EpCAM antibody (H) for 60 min. Alternatively, stem cell organoid–derived starved IECs were preincubated with C1q (F) or annexin V (G) for 15 min. After 2 h coculture of pretreated cell monolayers with untreated starved IECs (D, E, and H) or untreated cell monolayers with pretreated starved IECs (F and G), efferocytosis events (TR^+^ cells) were recorded in 20–40 image fields of the size 1.25 × 1.00 mm obtained in two independent experiments. Mann–Whitney U test. ***, P < 0.001; ****, P < 0.0001; ns, non-significant.

Mechanistically, efferocytosis is mediated by so-called “eat-me” signals, adapters, and receptors (Doran et al., 2020; Boada-Romero et al., 2020; Trzeciak et al., 2021). To gain insight into the expression of potential signals, adapters, and the presence of receptors on the intestinal epithelium, we next analyzed the intestinal epithelial transcriptome. A comparative analysis of the transcriptional profile of the neonatal (3 days after birth) versus adult (21 days after birth) small intestinal epithelium revealed an increase in the expression of the genes encoding the “eat-me” adaptors milk fat globule-EGF factor 8 protein (Mfg-E8), thrombospondin (THBS)1, growth arrest–specific (Gas)6 and C1q as well as of the receptors CD36, platelet and endothelial cell adhesion molecule 1, T cell membrane protein (Tim)4, and integrin-αv (CD51) in the newborn host (Fig. 3 A; GEO GSE35596; Pott et al., 2012). The expression of the genes for integrin-αv (CD51) and CD36 in the neonatal intestinal epithelium was further enhanced upon infection with *S.* Typhimurium (Fig. 3 B; GEO GSE51160; Zhang et al., 2014). RNA sequencing (RNA Seq) of flow cytometrically sorted *S.* Typhimurium–positive (*Salmonella*^+^) and –negative (*Salmonella*^−^) enterocytes from *S.* Typhimurium–infected neonatal mice on day 4 p.i. and comparison with total IECs from age-matched uninfected animals revealed upregulation of THSB1 specifically in *S.* Typhimurium–positive cells and increased expression of CD36 and integrin-αv (CD51) by both *S.* Typhimurium–positive and –negative cells (Fig. 3 C; GEO GSE248674; Fulde et al., 2021). To test the functional relevance of individual signal-receptor interactions, we next used inhibitory antibodies, signal inhibitors, or receptor antagonists of efferocytosis in our stem cell organoid co-culture model (Monks et al., 2005). Antibody-mediated blocking of both CD36 and integrin-αv (CD51) significantly reduced the number of internalized tdTomato-positive starved IECs or cell fragments in neonatal stem cell organoid monolayers (Fig. 3, D and E). Inhibition of surface-exposed phosphatidylserine by preincubation of the starved IEC with annexin V or inhibition of the C1q receptor by preincubation of the neonatal stem cell–derived cell monolayer with C1q prior to coculture also significantly reduced the number of efferocytosis events (Fig. 3, F and G). In contrast, coculture in the presence of an epithelial cell adhesion molecule (EpCAM)–binding antibody did not affect efferocytosis efficacy (Fig. 3 H). These results suggest that the “eat-me” signals, adapters, and receptors phosphatidylserine, C1q, integrin-αv, and CD36 contribute to enterocyte efferocytosis.

**Figure 4.**
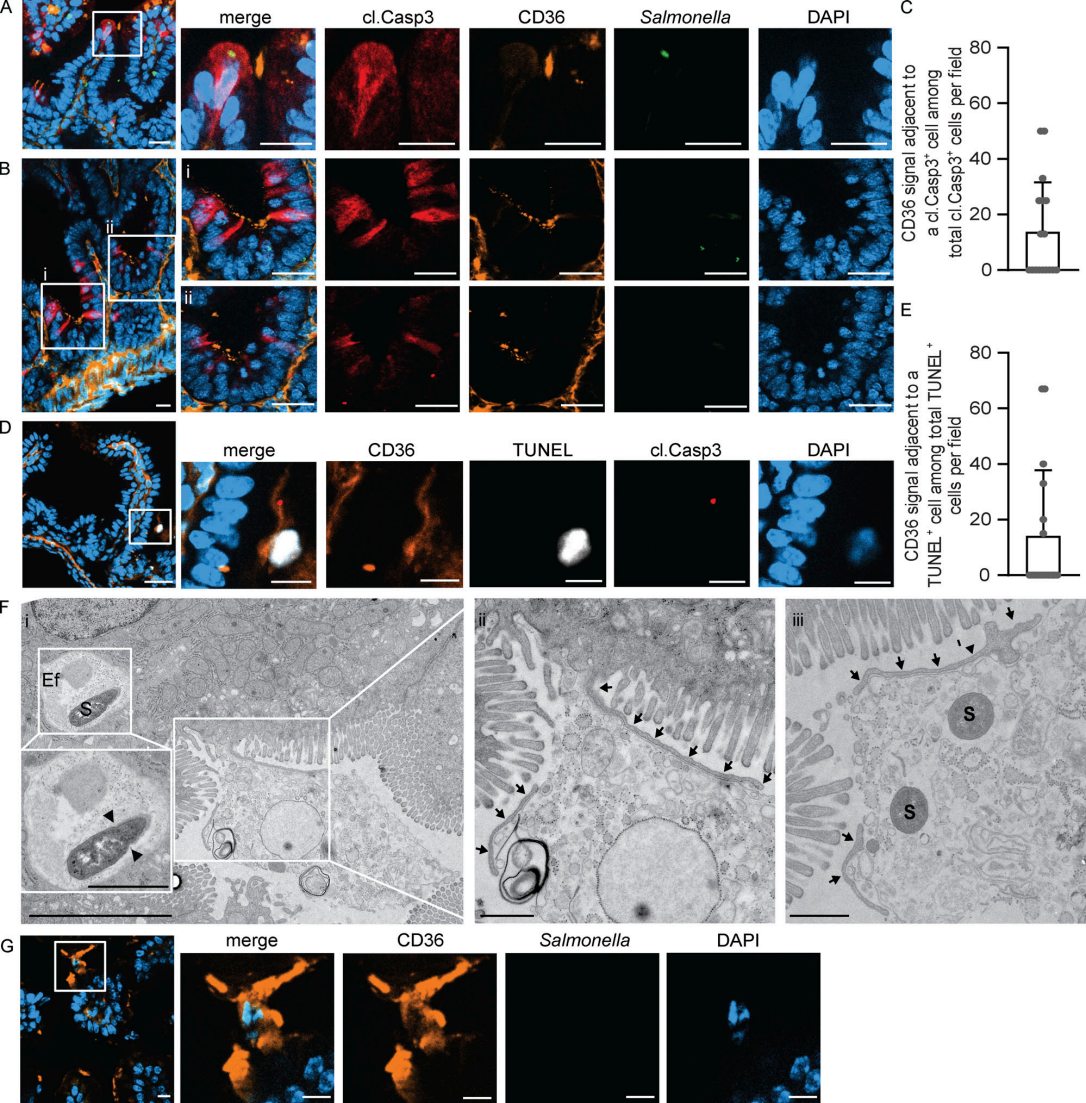
**Visualization of enterocyte efferocytosis in the neonatal intestine in vivo.** Immunostaining and TEM of small intestinal tissue sections of *S.* Typhimurium–infected neonate mice at day 4 p.i. **(A and B)** Immunostaining for cleaved caspase 3 (cl. Casp. 3, red), CD36 (orange), and *S.* Typhimurium (green). Focal CD36 expression by epithelial cells of the opposite villus (A) or by neighboring cells within the epithelial cell layer (B) of cleaved caspase 3–positive and in A also *S.* Typhimurium–positive IECs. Counterstaining with DAPI (blue). Bar, 20 μm. **(C)** Number of cleaved caspase 3 (cl. Casp 3)–positive cells with an adjacent CD36 signal among all cl. Casp 3–positive cells. **(D)** Immunostaining for CD36 (orange), TUNEL (white), and cleaved caspase 3 (cl. Casp3, red). Counterstaining with DAPI (blue). Bar, 20 μm (overview image) and 10 μm (single-color channels). **(E)** Number of TUNEL-positive cells with an adjacent CD36 signal among all TUNEL-positive cells. **(C and E)** 21 image fields with the size of 312.35 × 250.61 µm of small intestinal tissue sections from four individual *S.* Typhimurium–infected neonatal animals at day 4 p.i. were analyzed. **(F)** TEM images showing long membrane protrusions extending from the apical plasma membrane of the epithelium (arrows) engulfing luminal cell debris. Arrows in the left inset in panel i indicate the septum of a dividing *S.* Typhimurium (S) within an efferosome (Ef). Panel ii is a higher-resolution image of the area indicated in panel i. Two bacteria (S) are situated in the lumen within cell debris (iii). Panel iii was imaged on a serial section through the same tissue block. Bar, i = 5 μm, ii and iii = 1 μm. **(G)** Immunostaining for CD36 (orange) and *S.* Typhimurium (green). Counterstaining with DAPI (blue). Bar, 10 μm.

Having identified the “eat-me” adapters and receptors involved, we next set out to visualize the process of enterocyte efferocytosis in the neonatal gut. Staining for the efferocytosis receptor CD36 and cleaved caspase 3 as early markers for potentially still reversible innate immunity-induced cell death (Hornik et al., 2016) revealed focal CD36 expression on enterocytes adjacent to cleaved caspase 3–positive epithelial cells (Fig. 4, A and B). CD36 staining was observed on enterocytes at the opposite villus of the cleaved caspase 3–positive *S.* Typhimurium–positive epithelial cell (Fig. 4 A) or on neighboring enterocytes within the cell layer (Fig. 4, B and C). In contrast, epithelial CD36 expression was low in the small intestine of age-matched healthy neonates (Fig. S3 C). In some cases, TUNEL-positive enterocytes were found sloughed off within the intestinal lumen, and again, enhanced CD36 staining was detected at the apical plasma membrane of the epithelial cells immediately adjacent to the sloughed-off cell (Fig. 4, D and E). Given the potentially very transient nature of this scenario (prior to uptake of the cleaved caspase 3– or TUNEL-positive cell by efferocytosis), we believe that a percentage of 15–20% of cells with adjacent CD36 signal is substantial (Fig. 4, C and E). We also visualized *S.* Typhimurium–containing enterocytes showing morphological signs of cell death and protruding from the epithelial cell layer by electron microscopy in vivo (Fig. S3 D). Electron microscopic imaging of small intestinal tissue sections from *S.* Typhimurium–infected animals captured the likely process of enterocyte efferocytosis by IECs in vivo. Similar to the ultrastructural visualization of the stem cell organoid coculture suggesting massive plasma membrane rearrangement during the process of efferocytosis (Fig. 2, E and F), large membrane protrusions were observed extending from the structurally intact epithelial surface and adhering to and surrounding luminal cell debris (Fig. 4 F). Remarkably, similar membrane protrusions were also observed by immunofluorescence and these structures specifically stained positive for the efferocytosis receptor CD36 (Fig. 4 G). Thus, non-professional efferocytosis of luminal and adjacent enterocytes could be visualized in the small intestine of *S.* Typhimurium–infected animals in vivo and was associated with massive membrane rearrangements and the efferocytosis receptor CD36.

The observed non-professional efferocytosis is in striking contrast to the previously demonstrated mechanism of rapid exfoliation and luminal shedding of *S.* Typhimurium–positive colonic enterocytes in adult infected animals (Sellin et al., 2014; Hausmann et al., 2020; Fattinger et al., 2021). Recognition of intracellular bacteria by the cytosolic Naip1-6/Nlrc4 inflammasome induced controlled cell expulsion into the intestinal lumen. This limited *Salmonella* replication at the intestinal mucosa, prevented systemic dissemination of the pathogen, and reduced inflammation-associated tissue damage (Sellin et al., 2014; Fattinger et al., 2021; Hausmann et al., 2020). Notably, enterocyte exfoliation following innate immune stimulation or infection was less commonly observed in the neonatal intestine also in other models (Sellin et al., 2014; Zhang et al., 2014, 2018; Hughes et al., 2020). The underlying reasons are not understood, but exfoliation of enterocytes may release innate immune stimuli and induce inflammation or inappropriately reduce the absorptive surface, compromising enteral nutrition during the metabolically highly vulnerable neonatal period (Blander, 2016; Parker et al., 2019). Moreover, the hypometabolic state and energetic tradeoffs of the neonatal host during infection may not allow for the loss of the cell-bound anabolic substrates and the reduction of the enteric absorptive surface (Ganeshan et al., 2019; Green et al., 2016). In addition, cell exfoliation may not be possible due to the absence of crypt-based, rapidly proliferating progenitor cells, resulting in a reduced proliferative capacity and therefore limited ability of the murine neonatal epithelium to replenish the intestinal surface barrier (de Santa Barbara et al., 2003; Blander, 2016). Finally, enterocyte exfoliation has been shown to influence the composition of the enteric microbiota with potentially adverse consequences during the establishment of the enteric microbiota in the neonatal host (Anderson et al., 2021). Thus, non-professional efferocytosis and exfoliation may represent two alternative mechanisms to remove stressed or damaged cells and restore intestinal epithelial barrier integrity in the challenged neonatal and adult host, respectively. Notably, although non-professional efferocytosis was largely absent in adult *S.* Typhimurium–infected animals, professional efferocytosis mainly by macrophages and dendritic cells has been described in adult animals to contribute to the removal of villus enterocytes under physiological conditions (Cummings et al., 2016). In addition, non-professional efferocytosis is an important mechanism during embryogenesis, and therefore the observed phenotype in neonatal mice may reflect the early developmental state of the newborn host (Hoijman et al., 2021). In contrast, reduced clearance of senescent neutrophils by efferocytosis in the neonatal spleen and liver has recently been shown to increase the opsonophagocytotic capacity and age-dependent resistance to *Streptococcus pneumoniae* infection (Bee et al., 2023).

Surface exposure of the phospholipid phosphatidylserine (PtdSer) is the most widely studied and universal “eat-me” signal (Fadok et al., 1992, 1998, 2001). PtdSer is recognized by a wide variety of receptors including TIM4, brain-specific angiogenesis inhibitor (BAI)1, stabilin 2, CD300 family members, integrins via Mfg-E8, and by the TAM receptors Tyro3, Axl, and Mer via Gas6 (Hochreiter-Hufford and Ravichandran, 2013). In addition, we identified involvement of complement component C1q in intestinal epithelial efferocytosis in the neonatal host and signaling via CD91 (Ogden et al., 2001). The “eat-me” receptor CD36 and the integrin-αv (CD51) also contributed significantly suggesting cooperative binding of damaged enterocytes via the multidomain molecule THBS1 (Savill et al., 1990, 1992). Thus, multiple signal-receptor pairs such as surface-exposed PtdSer and its many potential receptors, C1q and CD91, as well as THSB1 and integrin-αv (CD51)/CD36 appear to cooperatively facilitate non-professional efferocytosis at the neonatal intestinal epithelium like that described for professional efferocytosis.

Efferocytosis by professional phagocytes exerts an overall protective effect consistent with its homeostatic and anti-inflammatory influence. Several studies have shown that animals deficient in critical signal and receptor molecules have an adverse outcome in intestinal models of inflammation. For example, the macrophage G protein-coupled receptor G2A enhances efferocytosis of dying granulocytes, and deletion of G2A increases dextran sulfate sodium (DSS)–induced colitis (Frasch et al., 2011, 2016). Similarly, Mfg-E8- and Gas6-deficient mice showed increased inflammation and reduced wound healing in a DSS colitis model (Kusunoki et al., 2015; Akitake-Kawano et al., 2013). Loss of efferocytosis receptor integrin-αv (CD51) expression on myeloid cells impaired the generation of mucosal T regulatory cells, leading to spontaneous colitis (Lacy-Hulbert et al., 2007). Similarly, loss of the TAM receptors Axl and Mer, expressed by radioresistant mucosal macrophages, reduced the clearance of apoptotic neutrophils and increased cytokine expression and inflammation in the colon (Bosurgi et al., 2013). However, less is known about non-professional efferocytosis in the context of intestinal tissue injury. Only one study reported that mice deficient in the PtdSer-recognizing receptor BAI1 exhibited more severe DSS colitis and that transgene expression by colonic epithelial cells was sufficient to rescue this phenotype (Lee et al., 2016). Thus, professional efferocytosis is an established mechanism of mucosal tissue homeostasis.

The functional consequences of non-professional enterocyte efferocytosis during infection remain unclear. Attempts to inhibit enterocyte efferocytosis in vivo by blocking a single eat-me receptor have so far failed, likely as a result of their redundant function (data not shown). The observation that neonatal *S.* Typhimurium–infected MyD88^−/−^ mice had significantly reduced enterocyte efferocytosis, but a similar bacterial organ count suggests that innate immune stimulation may directly or indirectly promote efferocytosis (Fig. S3, E–G). Enterocyte efferocytosis occurred after infection with invasive *S.* Typhimurium but not with adherent enteropathogenic *E. coli* (data not shown), suggesting that some type or degree of immune stimulation of lamina propria immune cells induced by invasive infection may be required (Dupont et al., 2016). Finally, the presence of type II DNase activity in efferosomes indicated efferosome-lysosomal fusion, a process actively inhibited by *S.* Typhimurium in classical SCVs, and suggested that efferocytosis may be associated with some degree of antimicrobial activity, consistent with a previous report (Capasso et al., 2016).

On the other hand, the majority of bacteria in the ultrastructural visualization showed no evidence of damage to the bacterial cell wall and identified actively dividing bacteria in efferosomes (inset in Fig. 1, D ii, inset in Fig. 4, F i). This is consistent with observations made during infection with other pathogens. For example, *Mycobacterium tuberculosis* resists lysosomal fusion in neutrophils and uses efferocytosis by macrophages to facilitate silent uptake, intracellular growth, and persistent infection (Dallenga et al., 2017). Also, *Salmonella* has been shown to actively induce efferocytosis of infected macrophages by neutrophils (Hiyoshi et al., 2022). In contrast to exfoliation, enterocyte efferocytosis allowed intracellular *S.* Typhimurium to remain in an intact intracellular niche, which could ultimately facilitate invasive infection. Efferocytosis has also been shown to exert a direct anti-inflammatory effect through the secretion of immunomodulatory cytokines such as prostaglandin E_2_ and transforming growth factor β (Juncadella et al., 2013; Sandahl et al., 2010; Lee et al., 2017), and this anti-inflammatory activity may reduce the antimicrobial response of the infected tissue (Caruso et al., 2012; Dutta et al., 2022). Enterocyte efferocytosis may therefore help to control the mucosal host response to *S.* Typhimurium in the neonatal host (Zhang et al., 2014, 2018; Dejani et al., 2018). Finally, remnants of the former host cell might provide a valuable source of nutrients (Anderson et al., 2021). Thus, although *S.* Typhimurium appears to evade the antimicrobial activity in enterocyte efferosomes and may benefit during pathogenesis, efferocytosis may still provide protection against other, less pathogenic microorganisms, and future studies are needed to clarify this issue.

In conclusion, we demonstrate non-professional efferocytosis by enterocytes as a unique feature of the infected neonatal small intestine. While professional efferocytosis is well established, non-professional efferocytosis of enterocytes by IECs has not previously been described in the context of infection. This novel feature of the neonatal intestinal epithelium may have important implications for host–microbial interaction and the antimicrobial host defense against many enteropathogenic microorganisms. It also highlights the unique situation of the neonatal gut and its ability to cope with the high and sometimes competing demands that accompany this metabolically and immunologically challenging period of life.

## Materials and methods

### Animal experiments

*S.* Typhimurium ATCC 14028 (NCTC12023) carrying a GFP expression plasmid (AmpR, kindly provided by Brendan Cormack, Stanford University, Stanford, CA, USA) and *S.* Typhimurium ATCC 14028 carrying an mCherry expression plasmid (AmpR, kindly provided by Leigh Knodler, National Institutes of Health, Hamilton, MT, USA) were cultured overnight at 37°C in Luria Bertani (LB) broth. Overnight cultures were diluted 1:10 in fresh LB medium and incubated at 37°C until reaching the logarithmic growth phase (OD_600_ ∼0.5). Bacteria were washed and adjusted to OD_600_ 0.55–0.60 containing 1.5–2.0 × 10^8^ CFU/ml. Experiments with neonatal mice were performed on 1-day-old C57BL/6 animals with a visible milk spot confirming a lactating dam or 6-day-old animals. Neonates were infected orally with 10^2^ CFU *S.* Typhimurium in 1 μl PBS. The inoculum given was confirmed by serial dilution and plating. Oral infection of 4–6-wk-old adult mice was performed as previously described (Barthel et al., 2003). Infection and control animals were derived from the same animal facility and hygiene barrier (Institute of Animal Science, RWTH University Hospital). Small intestinal tissues were fixed in 4% paraformaldehyde (PFA) for 20 min or overnight depending on the subsequent analysis. Intestinal crypts were isolated as described below.

### Ethics statement

Neonatal and adult C57BL/6 wild-type and Rosa^mT^/^mG^ transgenic (stock no. 007576; Jackson Laboratory) and B6.129P2(SJL)-MyD88tm1.1Defr/J (MyD88^−/−^, stock no. 009088, Jackson Laboratory) mice were housed under specific pathogen–free conditions and maintained on a 12-h light and dark cycle with food and water ad libitum according to the guidelines of the Federation for Laboratory Animal Science Associations and the German Society of Laboratory Animal Science (https://www.gv-solas.de). All animal experiments were performed in accordance with the German Animal Welfare Act (TierSchG) and approved by the local animal care committees, the Niedersächsische Landesamt für Verbraucherschutz und Lebensmittelsicherheit Oldenburg, Germany (approval 12/0697, 12/0693, 13/1097, and 14/1385), and the Landesamt für Natur, Umwelt und Verbraucherschutz, North Rhine Westfalia (84-02.04.2017.A397 and 40152A4).

### Intestinal epithelial stem cell organoid culture

Small intestinal crypts were isolated from the total small intestine of 1-day-old (Rosa^mT/mG^, C57BL/6) mice or from the middle part (jejunum) of the small intestine of 6-wk-old (Rosa^mT/mG^, C57BL/6) mice by incubation for 5 min at 4°C in PBS containing 2 mM EDTA as previously described (Kayisoglu et al., 2021). Isolated crypts were embedded in Matrigel (356231; BD Biosciences) and seeded into 48-well plates (20 μl of Matrigel per well). The Matrigel was polymerized for 15 min at 37°C and 250 μl of ENR basal culture medium (advanced DMEM/F12 medium [12634-028; Gibco] supplemented with penicillin/streptomycin [15140-122; Gibco], 0.01 M HEPES [15630-056; Gibco], 1× Glutamax [35050-038; Gibco], 1× N2 [17502-048; Gibco], 1× B27 [17504-044; Gibco], 500 mM N-acetylcysteine [A9165; Sigma-Aldrich], 50 µg/ml mouse EGF [PMG8045; Gibco], 100 µg/ml mouse noggin [250-38; PeproTech], and 10% of R-spondin conditioned medium purified from the supernatant of stably transfected HEK293T cells, kindly provided by Calvin Kuo, Stanford University, Stanford, CA, USA) was added per well. Fresh medium was added every 3 days and organoids were passaged at a 1:5 split ratio after 7 days. To obtain stem cell organoid–derived epithelial cell monolayers, 4-day-old spherical stem cell organoids prepared from neonatal or adult mice were trypsinized with TryLEExpress (12605-010; Gibco). After filtering and washing the cells by centrifugation, cell pellets were resuspended in ENRWY medium (ENR medium containing 50% Wnt3a conditioned medium) purified from the supernatant of a stably L-Wnt-3A expressing cell line (kindly provided by Sina Bartfeld, University of Würzburg, Würzburg, Germany) and 10 µM RhoK inhibitor Y-27632 (M20999; AbMole Bioscience). 200 μl of cell suspension was added to the Matrigel-coated glass surface of 8-well chamber slides or the plastic surface of 48-well cell culture plates followed by a 1-min centrifugation step to allow the cells to approach the Matrigel layer. After 16–18 h incubation at 37°C, non-adherent cells were removed with prewarmed PBS. The cells were then incubated again at 37°C for a further 24 h. Before using the cell monolayer, dead cells were removed with prewarmed PBS.

### Stem cell organoid co-culture assay

Stem cell organoids generated from neonatal or adult small intestinal tissue of Rosa^mT/mG^ mice were starved of essential growth factors by culturing in an advanced DMEM/F12 medium for 48 h. Starved spheroid stem cell organoids were trypsinized in TryLEExpress (12605036; Gibco). After washing, starved tdTomato IECs were added to adult or neonatal stem cell organoid cell monolayers. The cultures were centrifuged for 30 s to allow the tdTomato IECs to approach the stem cell organoid cell monolayer. After 2 h incubation at 37°C, cells were washed with prewarmed PBS to remove non-attached cells, and cell layers were fixed, stained, and analyzed by fluorescence microscopy. To evaluate the contribution of individual eat-me receptors, adapters, or signals, neonatal intestinal epithelial stem cell organoid cell monolayers were incubated with 100 µg/ml of purified mouse anti-mouse CD36 antibody (clone CRF D-2712; BD Pharmingen), purified isotype antibody (clone M18-254; BD Pharmingen), purified rat anti-mouse integrin-αv (CD51) antibody (clone RMV-7, 550024; BD Pharmingen), anti-mouse CD326 (EpCam) antibody (clone G8.8, 118210, BioLegend), or purified isotype antibody (clone R3-34; BD Pharmingen) for 60 min at 37°C. After the incubation, free antibody was removed by washing with prewarmed PBS. Similarly, growth factor–starved stem cell organoid cells generated from adult small intestinal tissue of Rosa^mT/mG^ mice were incubated with 25 µg/ml mouse C1q (CompTech complement Technology M009) or 200 µg/ml Annexin V (640901; BioLegend) for 15 min to block binding sites. After incubation, free protein was removed by washing with prewarmed PBS. Subsequently, starved IECs were co-cultured with the organoid cell monolayers for 2 h. The cell monolayers were then washed with prewarmed PBS, fixed, stained, and analyzed by fluorescence microscopy.

### Immunostaining

Immunostaining was performed on distal small intestinal tissue in both adult and neonatal animals. 4 μm PFA-fixed paraffin-embedded tissue sections were deparaffinized in xylene and rehydrated followed by antigen retrieval in 10 mM sodium citrate. Tissue sections were blocked with 10% normal donkey serum in 5% bovine serum albumin (BSA)/PBS. 5 μm frozen tissue sections were fixed in methanol at −20°C for 20 min prior to the blocking step. Chicken anti-GFP (ab13970; Abcam), rabbit anti-*Salmonella* O4 antigen (ab35156; Abcam), rat anti-Lamp1 (1D4B, Developmental Studies Hybridoma Bank, University of Iowa, USA), rabbit anti-cleaved caspase-3 (#9661; Cell Signaling Technology), mouse anti-mouse CD36 (clone CRF D-2712; BD Pharmingen), and mouse anti-E-cadherin (610182; BD Transduction Laboratories) antibodies, and appropriate fluorophore-conjugated secondary antibodies (Jackson ImmunoResearch) were used for immunostaining. The ApopTag ISOL dual fluorescence apoptosis detection kit (APT1000; Millipore) was used to detect DNase type II cleavage activity. The In Situ Cell Death Detection kit TMR red (12156792910; Roche) was used to detect TUNEL-positive cells on tissue sections. PFA-fixed paraffin-embedded tissue sections required proteinase K treatment prior to TUNEL staining. Frozen tissue sections were permeabilized prior to TUNEL staining. For immunofluorescence analysis of efferocytosis by stem cell organoid cell monolayers, cells were fixed with 4% PFA and blocked with 5% BSA. MFP488 Phalloidin (MFP-A1379; MoBi Tec) and the Alexa Fluor 488–conjugated anti-mouse CD326 (EpCam) antibody (clone G8.8, 118210; BioLegend) were used to visualize the actin skeleton and epithelial cells, respectively. Alexa Fluor 647–conjugated wheat germ agglutinin (WGA, W32466; Invitrogen) was used to detect the mucus layer. Slides were mounted in DAPI mounting medium (H-1200-10; Vector) and images were captured using a Zeiss ApoTome.2 system microscope connected to an Axiocam 506 digital camera. Images were formatted using the ZEN 3.4 imaging software.

### Comparative gene expression analysis

The comparative transcriptome analysis of IECs from 3- versus 21-day-old mice has been previously reported and is accessible via GEO Series accession number GSE35596 (Pott et al., 2012). The comparative transcriptome analysis of IECs from neonatal *S.* Typhimurium–infected and age-matched healthy control animals was previously reported and is accessible through GEO Series accession number GSE51160 (Zhang et al., 2014). The comparative transcriptomic analysis of flow cytometrically sorted *S.* Typhimurium–positive and –negative IECs from *S.* Typhimurium–infected neonatal mice is based on a previously performed dual RNA Seq analysis and is accessible via GEO Series accession number GSE248674 (Fulde et al., 2021). Briefly, 1-day-old mice were orally infected with 10^2^ CFU *S.* Typhimurium constitutively expressing mCherry. IECs were isolated on day 4 p.i. as previously described (Zhang et al., 2014), and EpCam^+^ CD45^−^ cells were differentially sorted by flow cytometry for mCherry-positive (*S.* Typhimurium–infected) and mCherry-negative (uninfected) cells, similar to a previously established protocol (Frönicke et al., 2018). Total RNA was isolated from both cell populations using the QIAGEN RNeasy Micro Kit, and genomic DNA was removed by DNase I digestion. Due to the relatively low number of infected epithelial cells (∼1% of total epithelial cells), *S.* Typhimurium–positive cells from 49 neonates from seven litters had to be pooled to obtain sufficient RNA amounts for sequencing. cDNA libraries were generated according to Westermann et al. (2016). RNA Seq analysis was performed using READemption’s (Förstner et al., 2014, version 0.3.5; Förstner, 2015) sub-command “align” building on segemehl (Hoffmann et al., 2009, version 0.2.0) to generate read mappings and the sub-command “gene_quanti” to create gene-wise read countings. Those countings were used to conduct differential gene expression with DESeq2 (Love et al., 2014).

### Ultrastructural analysis

The distal small intestine of mice at day 4 p.i. was immersion fixed with 4% PFA in 200 mM HEPES, pH 7.4, overnight at room temperature. For resin embedding, the tissue was post-fixed with 1% glutaraldehyde (GA) overnight and with 1% osmium tetroxide prepared in 1.5% potassium ferricyanide/dH_2_O for 1 h on ice. Tissue was contrasted en bloc with 2% aqueous uranyl acetate for 2 h at room temperature. Dehydration was performed with an ethanol series 50–70–80–90–96–100–100%–100%–100%, each for a minimum of 15 min. Tissue was progressively infiltrated with Epon_812 substitute embedding resin (33–66–100%, each for minimum 12 h) and polymerized at 70°C for 2 days. Stem cell organoid co-cultures on glass coverslips were fixed by adding 2% GA in 200 mM HEPES directly to the medium at a 1:1 volume ratio. After 5 min, the medium was exchanged with 1% GA in the HEPES buffer. Samples were treated as described above except that acetone was used in addition for dehydration after the ethanol series. Coverslips were embedded upside down onto an EPON block. After polymerization, the glass was dissolved with 40% hydrofluoric acid. Ultrathin 80-nm sections were cut using a Leica UC7 ultramicrotome, deposited onto copper, slot, formvar-coated grids, and contrasted with saturated aqueous uranyl acetate for 10 min, and with Reynolds’ lead citrate stain for 3 min. Grids were imaged in a Tecnai G2 Spirit BioTWIN transmission electron microscope (FEI; now Thermo Fisher Scientific), operated with a LaB6 cathode at 80 kV, or with a JEM 1400 transmission electron microscope (JEOL) operated with a Tungsten filament at 120 kV. Images on the Tecnai microscope were taken with a CCD bottom-mount Eagle HS 4 k × 4 k camera, using TIA software (v. 2.5; all FEI/Thermo Fisher Scientific), or with the side-entry MegaView G2 camera using the iTEM software (v. 5.0; all EMSIS). Images on the JEOL microscope were taken with a CMOS bottom-mount TemCam F216 camera using the EM-Menu software (all TVIPS).

### ELISA

Neonatal and adult small intestinal epithelial stem cell organoid–derived cell monolayers were generated as described above. Cell monolayers were left untreated, infected with *S.* Typhimurium at a multiplicity of infection (MOI) of 10:1, or exposed to starved IECs, and incubated at 37°C for 2 h. Starved IECs and the *S.* Typhimurium inoculum were prepared as described above. The supernatants were collected and TNF was quantified according to the manufacturer’s instructions for the MAX Deluxe Set Mouse TNF-α ELISA (430915; BioLegend).

### Statistical analysis

The one-way ANOVA Kruskal–Wallis test with Dunn’s post-test and the Mann–Whitney U test were used for statistical analysis. The GraphPad Prism software 9.0 was used for statistical evaluation. P values are indicated as follows: ****, P ≤ 0.0001; ***, P < 0.001; **, P < 0.01; *, P < 0.05; ns, P > 0.05.

### Online supplemental material

Additional imaging data and quantitative results on enterocyte efferocytosis can be found in Figs. S1, S2, and S3.

## Data Availability

The data underlying Fig. 3 are openly available in Gene Expression Omnibus NCBI (GEO) at https://www.ncbi.nlm.nih.gov/gds with the accession numbers GSE35596, GSE51160, and GSE248674. The bioinformatic analysis workflow of the dual RNA Seq dataset was deposited at https://doi.org/10.5281/zenodo.13926.
